# Social differences in mortality and life expectancy in Germany. Current situation and trends

**DOI:** 10.25646/5872

**Published:** 2019-03-14

**Authors:** Thomas Lampert, Jens Hoebel, Lars Eric Kroll

**Affiliations:** 1 Robert Koch Institute, Berlin Department of Epidemiology and Health Monitoring; 2 Formerly Robert Koch Institute, Berlin Department of Epidemiology and Health Monitoring

**Keywords:** SOCIAL INEQUALITY, SOCIOECONOMIC STATUS, INCOME, MORTALITY, LIFE EXPECTANCY

## Abstract

Social differences in mortality and life expectancy are a clear demonstration of the social and health-related inequalities that exist within a particular population. According to data from the Socio-Economic Panel (SOEP) for the period ranging from 1992 to 2016, 13% of women and 27% of men in the lowest income group died before the age of 65; the same can be said for just 8% of women and 14% of men in the highest income group. The difference between mean life expectancy at birth among the lowest and highest income groups is 4.4 years for women and 8.6 years for men. Substantial differences also exist between income groups regarding further life expectancy at the age of 65: women in the lowest income group have a 3.7-year shorter life expectancy than women in the highest income group. Similarly, men in the lowest income group have a 6.6-year shorter life expectancy than men in the highest income group. Finally, results from the trend analyses suggest that social differences in life expectancy have remained relatively stable over the last 25 years.

## 1. Introduction

Germany is not only one of the wealthiest countries in the world, it also has well-developed social security and pension systems. However, significant inequalities continue to exist in terms of people’s living conditions and the opportunities that people have to participate in society. These factors are reflected in a highly unequal distribution of income and wealth, poor prospects for low-skilled people in the labour market, growth in precarious employment and the continuing close links between social status and educational opportunities in Germany [1].

Social inequality is important from a public health and health policy perspective because it has an impact both on the population’s health and life expectancy. People on low incomes or with a low occupational or educational status are at increased risk of developing chronic diseases and disorders. The same applies to illness-related functional limitations in everyday life and they also tend to have a lower general quality of life. Moreover, significant social differences also exist when it comes to individual health-related behaviour and behavioural risk factors such as smoking, physical inactivity, obesity and hypertension. Ultimately, these risks accumulate and are reflected in the higher premature mortality and shorter lifespans found among socially disadvantaged populations [2-4].

Analysis of social differences and their relation to mortality and life expectancy, therefore, is crucial. Importantly, the Socio-Economic Panel (SOEP) provides a possible empirical basis for this type of research. The SOEP is an annual household survey conducted by the German Institute for Economic Research (DIW) aimed at providing a current assessment of political and social change in Germany. A mortality follow-up (an identification of the reasons why study participants who took part in previous survey waves no longer participate) can be used to identify deaths among former participants. Numerous analyses of social differences in mortality and life expectancy have already been conducted using SOEP data. Most of these studies have focused on differences between income groups, although some have also looked into differences linked to educational and occupational status. They all identified significant social differences in mortality and life expectancy which were to the detriment of people with a low level of income, education or occupational status [5-10].

In the following section, SOEP data [11] are used to analyse income-related differences in mortality and life expectancy for the period ranging from 1992 to 2016. In addition to mean life expectancy at birth, this study also considers further life expectancy at the age of 65 and provides results from trend analyses. The trend analyses indicate whether and, if so, the extent to which social differences in mortality and life expectancy have changed over the past 25 years.

## 2. Methodology

Income-related differences in mortality and life expectancy can be studied using SOEP data collected from 83,287 study participants between 1992 and 2016. Not all of the individuals who provided data were studied for the entire period. On average, the study participants were observed for 7.4 years, with the data consisting of 617,550 one-year study episodes. A total of 4,193 study participants died during the study period (5.0% of the participants).

This study uses ‘net equivalised income’ as an indicator of income. Net equivalised income takes the size and composition of a particular household into account. This helps ensure that the savings linked to the shared economy of multi-person households and the different income needs of adults and young people are considered, while also providing for a comparison of household incomes despite differences in household size and age structure. Calculations of net equivalised income are based on a household’s net income, in other words, the total income of all household members after tax and social security contributions have been deducted. Equivalence balancing or needs weighting was undertaken in line with the new OECD equivalence scale, which is also used in official social and poverty monitoring. According to this scale, all persons aged 15 and over have an income need 0.5 times that of the head of the household and all children and adolescents under 15 have an income need 0.3 times that of the head of the household. Furthermore, the calculation uses a quotient based on the sum of the household members’ needs weighting (e.g. 1 for a one-person household; 1.5 for a household with two adults; and 2.1 for a household consisting of either two adults and two children or adolescents under the age of 15).

Between 1992 and 2016, the median net equivalised income of the population in Germany was €1,495.00. This figure was used to define the following five income categories: an income of less than 60%, of between 60% and under 80%, of between 80% and under 100%, of between 100% and under 150%, and an income of 150% or more of this figure. In accordance with socio-political definitions, households with an income of less than 60% of the median income, in other words, less than €897.00 can be described as affected by or as at risk of poverty. In contrast, the 150% threshold (€2,243.00) can be used to delineate relative wealth.

Analyses of income-related differences in life expectancy tend to use a method that combines the figures on relative mortality risks gained from survey data with those on the general risk of mortality identified from official period life tables [12]. This study calculated the relative risk of mortality by applying Cox regression models to the SOEP data. The results are differentiated according to time period, age group and a participant’s gender. In contrast to previous studies, instead of focusing on mortality risks for the entire range of ages reflected in the data, this study concentrated on specific age groups. This was done because the assumption that income remains constant during a person’s entire life was considered inaccurate. However, the limited number of cases available only meant that two age groups could be compared (people aged up to 50; and those aged 51 and above). Finally, a semi-parametric Cox model was used to prevent a priori assumptions about the relationship between age and mortality risk from influencing the results.

In order to gain a figure for mean life expectancy, mortality risks were extracted from the official life tables for Germany as provided by the Federal Statistical Office’s Genesis databank [13]. The databank provides annual figures that are structured according to age and gender. Until 2000, however, only abbreviated life tables are available with calculations based on a maximum age of 90 years. As calculations of life expectancy using mortality risks require complete life tables, mortality risks were extrapolated up to the age of 112, the age at which people were assumed to have a 100% mortality risk. In order to verify the results, these figures were then compared with the annual data available from the databank. The mean deviation between the figures used in this study and those provided by the Federal Statistical Office was less than 0.05 years for women and men at birth.

In order to gain baseline values for income group-specific survival rates and life expectancy, mean values for age- and gender-specific mortality risks were calculated using the official life tables for each respective study period. The relative mortality risks identified for the income groups in terms of the population average (calculated using SOEP data) were then applied to these baseline values while accounting for age and gender. Finally, the resulting income-specific rates were used to calculate survivor functions and life expectancy. In addition to average life expectancy at birth, this study also provides data on further life expectancy at the age of 65. Moreover, it also describes the proportion of women and men who died before reaching this age. All analyses were carried out using version 3.5 of the statistics package R [14]. Due to the complexity of the method applied, and to help ensure that the results could be reproduced, the procedure, the libraries and the functions developed for this study are documented in a ‘jupyter’ notebook [15]. Jupyter notebooks enable programs, results and comments to be saved in a single file. The relevant files have been made available on the online source code archive Github, which converts them into HTML pages so that they can be viewed with any web browser. However, they can also be downloaded, run or modified after installing the necessary runtime environment (Project Jupyter).

## 3. Results

**Figure 1:**
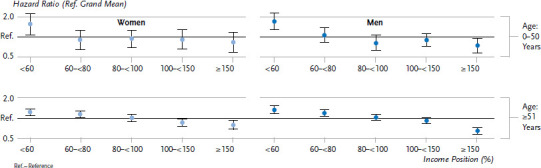
Relative mortality risks (hazard ratios) in relation to the average risk in SOEP (effect coding) according to gender, age group and income Source: SOEP 1992-2016

Women and men on an income that is below the poverty line had a significantly higher risk of mortality during the observation period than the population average. Income differences in mortality were somewhat more pronounced among women and men in the younger of the two age groups (people aged up to 50 years) than in the older group (51 years or above). Figure 1 shows the corresponding mortality risks in the ‘effect coding’ compared to the population average. However, instead of comparing the mortality risk faced by the lowest income group to the mean mortality risk of the population, these risks can also be compared to those faced by the highest income group. In this case, the mortality risk of women aged up to 50 in the low income group increases by a factor of 2.2, and it increases among men by a factor of 2.4. In the case of women and men aged 51 or above, the mortality risk increases by a factor of 1.5 and 1.9 respectively.

**Figure 2:**
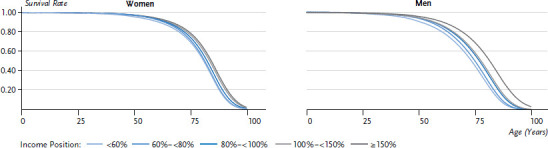
Survival rates according to gender and income Source: SOEP, period life table 1992-2016

The figures set out in Figure 1 were then applied to the mortality rates derived from the life tables. Survival rates for women and men in the five income groups demonstrate the proportion of women and men in each group who survived up until a certain age (Figure 2). As Figure 2 contains probabilities, a value of 0.75 means that 75% of the group was still alive at this point. From the age of 40, the lines in the men’s graph deviate, indicating that a larger proportion of men in the lower income groups had already died by this point compared to men in the higher income groups.

**Figure 3:**
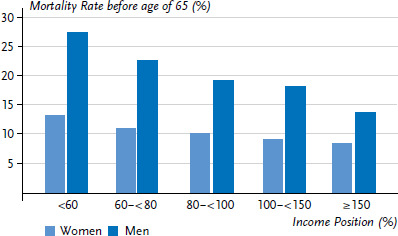
The proportion of women and men who die before the age of 65 according to income Source: SOEP, period life table 1992-2016

In order to emphasise this correlation more clearly, Figure 3 shows the proportion of women and men in each of the income groups that died prematurely (before the age of 65). The graph demonstrates that the lower the income, the higher the premature mortality. Whereas 13.2% of women in the lowest income group died before the age of 65, the same can be said of just 8.3% of women in the highest income group. Moreover, premature mortality among men is significantly higher across all income groups and the difference between the lowest and highest income groups is larger among men at 27.2% compared to 13.6%.

The mean life expectancy at birth for the period between 1992 and 2016 was 80.8 years among women and 75.0 years among men (Table 1). There was a 4.4-year and an 8.6-year difference between the lowest and highest income groups among women and men respectively. On average, women and men who reached the age of 65 could expect to live for 17.0 (women) and 12.5 (men) more years. A comparison of the lower and upper end of the income spectrum identified a 3.7-year difference in further life expectancy at the age of 65 among women, and a 6.6-year difference among men.

**Figure 4:**
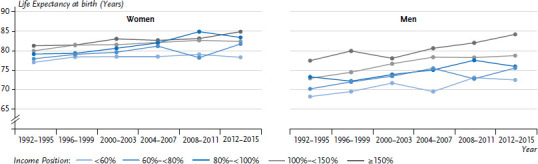
Trends in mean life expectancy at birth according to gender and income between 1992 and 2016 Source: SOEP, period life table 1992-2016

The results of the trend analysis show that mean life expectancy at birth during the 25-year observation period increased among women from 78.9 years to 82.2 years and among men from 72.3 years to 77.4 years. Increased life expectancy was observed among all income groups (Figure 4). However, life expectancy among women in the lowest income group rose by 1.4 years compared to 3.9 years among women in the highest income group. Similarly, men in the lowest income group saw their life expectancy rise by 4.2 years, whereas those in the highest income group gained 6.9 years. The increase in life expectancy in the three middle income groups varies between 2.6 and 4.6 years among women and between 2.6 and 5.9 years among men.

**Figure 5:**
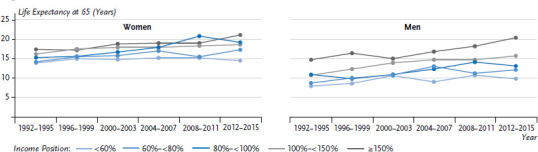
Trends in further life expectancy at the age of 65 according to gender and income between 1992 and 2016 Source: SOEP, period life table 1992-2016

During the observation period, further life expectancy at the age of 65 increased by 2.8 years among women and by 3.7 years among men. However, women in the lowest income group gained just 0.6 years compared to 3.7 years among women in the highest income group (Figure 5). Similarly, men in the lowest income group saw their life expectancy rise by 1.8 years, whereas life expectancy among men in the highest income group rose by 5.7 years. Women in the middle income groups gained an increase in further life expectancy at the age of 65 of between 2.4 and 3.9 years, men gained between 2.2 and 5.0 years of further life expectancy.

## 4. Discussion

Significant income-related differences in mortality and life expectancy were identified among men and women from analyses of SOEP data covering 1992 to 2016. A 4.4-year difference in mean life expectancy at birth was identified between women in the lowest and highest income groups over the entire period. Among men, this difference was 8.6 years. Further life expectancy at the age of 65 also differs between the lowest and highest income groups with 3.7 years among women and 6.6 years among men. The trend analysis found no reduction in these differences during the past 25 years. Rather, the results indicate that the increase in life expectancy during this period was higher in the highest and middle income groups. As such, the gap between the lowest and highest income groups may have increased during the study period. However, it was impossible to test for this statistically, as case numbers were relatively low and estimator uncertainty is particularly high in analyses focused on short spaces of time rather than entire observation periods. As this still needs to be kept in mind when considering the otherwise considerable leaps in life expectancy at birth that some income groups have experienced and in further life expectancy at the age of 65, these findings need to be viewed with caution.

The results of this study are broadly consistent with those of other studies of income-related differences in mortality and life expectancy. At the same time, they also apply to the results of previous studies that used data from the SOEP [6-9]; however, it is important to bear in mind that they are not always directly comparable with those of other studies. On the one hand, this study used a different observation period and a different methodology. Moreover, substantial changes have also been made to the data pool since these studies were conducted. The SOEP data is not derived from a static database; rather, each new data set can give rise to changes in the figures from previous years. This is due to missing values being entered at a later date, changes in imputation and weighting procedures and participant drop-out leading to fewer cases. In addition, this study made valid changes to the methodology, which could have influenced the results. Several of these changes were made to improve the stability of the results for trend analysis and to address limitations of the original approach [12]. Furthermore, this study provides estimates of age group-specific mortality risks, used Cox regression models, and relied on the information available on income at the beginning of each study period.

It is also important to note that no systematic follow-up is being undertaken in the SOEP as to reasons for non-participation. However, it is still possible to follow up and identify deaths that occurred before 2009 using a number of studies on attrition. No such studies have been carried out since then. As people in a poor state of health (who, therefore, also have a higher risk of death) drop out more frequently from studies like this, it is possible that mortality will be underestimated and life expectancy will be overestimated to a greater extent in the future [12]. Given that people on low incomes are more likely to be affected by ill health, this may also aggravate the uncertainty associated with estimates of income-related differences in life expectancy.

The results of this study are also consistent with studies that used other data sources. This includes the MONICA/KORA studies undertaken for the Augsburg region [16, 17], the life expectancy survey conducted by the Federal Institute for Population Research [18] and the German Health Interview and Examination Survey for Adults (DEGS1) [19], which also follow-up on their study participants. Analyses that demonstrate trends in differences in life expectancy, but, for example, examine the further life expectancy of individuals after a heart attack or of people with diabetes [20], have also identified differences related to income and other social indicators such as education and occupational status.

Studies based on data from Germany’s social insurers are also important, even though they face some additional limitations. For example, the validity of the data collected by statutory health insurers is limited due to the insurers’ selective membership structure [21]. In addition, their data on income often lacks information or is missing entirely, meaning that in most cases the educational and occupational status or sometimes a person’s type of insurance (compulsory versus voluntary insurance) are used instead. Be this as it may, there are some advantages in using data from statutory health insurers, such as the very large case numbers and the opportunity to carry out cause of death analyses. Findings from studies that used data from the AOK (Allgemeine Ortskrankenkasse) or the GEK (Gmünder Ersatzkasse) indicate significant social differences in mortality from heart attack, stroke and various cancers, including stomach, intestinal and lung cancer [21, 22].

A study based on data from the German Statutory Pension Insurance Scheme, which was restricted to male insurance holders, showed that a low income, as determined by earning points, was associated with a lower further life expectancy at the age of 65 years. In addition, the study indicated that income-related differences in further life expectancy continued to widen during the observation period (1995/1996 to 2007/2008). Although life expectancy increased among all income groups during this time, the increase was higher in high income groups than in lower income groups [23].

Comparable social differences in mortality and life expectancy have also been reported by studies using data from other countries, although most of them focused on education or occupational status; only very few concentrated on income. The results of a European research project that used data from nationwide health surveys and also undertook mortality follow-ups are particularly noteworthy. The results, which stem from 22 countries and are based on data from the 1990s and the 2000s, show that, on average, people with a low level of education have an approximately two-fold higher mortality risk across Europe than people with a high level of education. A study that took the various causes of death into account shows that these differences continue to exist in terms of cardiovascular and cancer-related deaths, as well as those caused by accidents and injuries. Moreover, a comparison of countries found that social differences in mortality were more pronounced in Eastern European countries than in Southern, Central and Northern European countries [24].

Studies have also been conducted of long-term developments for some other countries on social differences in mortality and life expectancy. In Europe, this particularly applies to the UK and Scandinavia. In the UK, data is available from the routine mortality follow-ups of the official census. A comparison of men and women in the lowest and highest occupational status groups in England and Wales using data that was gathered between 1982 and 1986 found a difference in mean life expectancy at birth of 3.8 years among women and 4.9 years among men. In the 20 years that followed, life expectancy in all status groups increased, as did the gap between the groups. Between 2002 and 2006, the difference was 4.2 years among women and 5.8 years among men [25].

The data for Norway also show that social differences in life expectancy have increased over the last few decades. This is illustrated by a study using data from the Norwegian National Register as well as population-based studies and databases compiled between 1961 and 2009. At the beginning of the 1960s, people with a low level of education aged 35 and above had an average further life expectancy of 44.1 years (women) and 40.3 years (men). The figures for women and men with a high level of education were 45.6 years and 42.2 years, respectively. By 2009, further life expectancy in the low education group had increased by 2.9 (women) and 2.1 years (men). The increase in life expectancy in the high education group was much higher at 6.1 (women) and 6.4 years (men) [26].

The social differences that exist in mortality and life expectancy pose a major challenge for public health and health policy [27, 28]. Further improvements to the data should be sought as a basis for continuous monitoring, which, in turn, is essential for planning, implementing and evaluating measures aimed at reducing social differences in mortality and life expectancy. Data from mortality follow-ups carried out as part of social scientific or health-related studies, as well as routine data from social insurance institutions, are available for Germany. Both empirical approaches are promising; each has its own methodological limitations [21, 29, 30].

Countries that have national mortality registries have a head start over Germany. Data from these registries can be combined with other data sources, such as nationally representative social science and health-related studies. Even if a comparable combination of different data sources would, at best, only partially be possible due to the data protection regulations in Germany, the establishment of a national mortality register would provide additional opportunities for analysis [31]. Moreover, a mortality register that offered information about the social situation of the deceased or that could be linked to data sources containing this information would significantly improve the basis for analysis of social differences in mortality and life expectancy and their associated developments over time.

## Key statements

- Social differences in life expectancy are an extreme indication of social inequality.
- About 13% of women and 27% of men on low incomes die before the age of 65.
- The difference in mean life expectancy at birth between the lowest and highest income group is 4.4 years for women and 8.6 years for men.
- At the age of 65, women in the lowest income group have a 3.7-year and men have a 6.6-year lower further life expectancy than women and men in the highest income group.

## Figures and Tables

**Table 1:** Mean life expectancy at birth and further life expectancy from the age of 65 according to gender and income Source: SOEP, period life table 1992-2016

	Mean life expectancy at birth (years)		Further life expectancy at the age of 65 (years)	
Income	Women	Men	Women	Men
<60%	78.4	71.0	15.2	9.8
60%–<80%	79.7	73.3	15.9	11.0
80%–<100%	80.7	75.2	16.9	12.4
100%–<150%	82.1	76.0	18.2	13.2
≥ 150%	82.8	79.6	18.9	16.4
Total	80.8	75.0	17.0	12.5
